# Shared phylogeographic patterns between the ectocommensal flatworm *Temnosewellia albata* and its host, the endangered freshwater crayfish *Euastacus robertsi*

**DOI:** 10.7717/peerj.552

**Published:** 2014-09-25

**Authors:** Charlotte R. Hurry, Daniel J. Schmidt, Mark Ponniah, Giovannella Carini, David Blair, Jane M. Hughes

**Affiliations:** 1 Australian Rivers Institute, Griffith University, Nathan, Qld, Australia; 2 School of Marine and Tropical Biology, James Cook University, Townsville, Qld, Australia

**Keywords:** Dispersal, Fragmented habitat, Haplotype sharing, Crustaceans, Comparative phylogeography, Headwater, Invertebrates

## Abstract

Comparative phylogeography of commensal species may show congruent patterns where the species involved share a common history. *Temnosewellia* is a genus of flatworms, members of which live in commensal relationships with host freshwater crustaceans. By constructing phylogenetic trees based on mitochondrial COI and 28S nuclear ribosomal gene sequences, this study investigated how evolutionary history has shaped patterns of intraspecific molecular variation in two such freshwater commensals. This study concentrates on the flatworm *Temnosewellia albata* and its critically endangered crayfish host *Euastacus robertsi*, which have a narrow climatically-restricted distribution on three mountaintops. The genetic data expands upon previous studies of *Euastacus* that suggested several vicariance events have led to the population subdivision of *Euastacus robertsi*. Further, our study compared historical phylogeographic patterning of these species. Our results showed that phylogeographic patterns shared among these commensals were largely congruent, featuring a shared history of limited dispersal between the mountaintops. Several hypotheses were proposed to explain the phylogeographic points of differences between the species. This study contributes significantly to understanding evolutionary relationships of commensal freshwater taxa.

## Introduction

There are many examples of commensal relationships between aquatic organisms, perhaps none more prevalent than in the relationship between crustacean hosts and Platyhelminthes. Both marine and freshwater crustaceans worldwide have been shown to have persistent infestations of Platyhelminthes flatworms (McDermott, Williams & Boyko, 2010; Ohtaka et al., 2012). However, not all of these associations are parasitic, many are commensal or mutualistic. An example of a commensal association is the one between the eastern Australian freshwater crayfish genus *Euastacus* and their ectocommensal temnocephalan flatworms. These flatworms are mostly host-specific and the most prevalent of just three, known, external symbionts on *Euastacus* (McCormack, 2012). Many temnocephalans are classified as free living, i.e., nonparasitic and capable of motility. They use the host purely as a mechanism to facilitate transport and/or feeding. The close association between the host and its ectocommensal may be exploited to develop an understanding of the phylogeographic history of both species.

Host-commensal associations can be examined using molecular data, which may demonstrate congruent patterns between host and commensal (Whiteman, Kimball & Parker, 2007; James et al., 2011). The correlation of genetic variation between interacting species may be linked to indirect factors such as shared responses to environmental heterogeneity (e.g., spatial dependence) or due to species sharing similar life histories and/or movement patterns (James et al., 2011). Hence, we can use genetic data to explore potential habitat boundaries, identify dispersal patterns, identify divergence events, discover cryptic gene flow or determine points of origin (Nieberding et al., 2004; Barbosa et al., 2012; Harris et al., 2013). For instance, Nieberding et al. (2004) explain how inferences can be made on host phylogeographic history by using the species which has the higher rate of molecular evolution (usually the symbiont) as a “biological magnifying glass”; i.e., the detection of previously unknown historical events of the host as derived from the phyleogeographic history of the symbiont. Vertical transmission in particular allows “parasites” to be used to infer genealogical history of the host (Rannala & Michalakis, 2003; Whiteman & Parker, 2005). An improved understanding of evolutionary relationships between taxa with closely dependent life-histories can lead to increased insight into phylogeographic patterns which may be an important factor when considering conservation management plans for endangered species (Whiteman, Kimball & Parker, 2007; Toon & Hughes, 2008). Further, phylogeographic histories are likely to track one another if the host exists in highly sub-divided populations, as is the case for many headwater species (McLean, Schmidt & Hughes, 2008; Hughes, Schmidt & Finn, 2009).

In this study we present the first comparative phylogeographic analysis of the ectocommensal flatworm *Temnosewellia albata* Sewell, Cannon & Blair, 2006 (Platyhelminthes, Temnocephalida, Temnocephalidae) and its critically endangered host crayfish *Euastacus robertsi* Monroe, 1977 (Arthropoda, Decapoda, Parastacidae); freshwater invertebrates of headwater streams. Our study seeks to understand the association between these commensals and is one of just a handful of phylogeographic studies of an ectocommensal flatworm. Our comparisons of phylogeographic histories were attained by sequencing the mitochondrial cytochrome oxidase subunit 1 (COI) and the nuclear 28S ribosomal DNA.

Previous studies have suggested the diverse array of *Euastacus* species in eastern Australia evolved through vicariance of formerly widespread ancestral taxa that became isolated in upland refuges of the eastern highlands during the Pliocene drying of the Australian continent (Ponniah & Hughes, 2004; Shull et al., 2005; Ponniah & Hughes, 2006). In these studies two species, *E. robertsi* and *E. fleckeri* were found to comprise a highly divergent monophyletic group within the genus. This phylogenetic separation of the two most northern *Euastacus* and the rest of the genus is coincident with a significant biogeographical barrier, the ‘Black Mountain Corridor’. Further, Ponniah & Hughes (2006) suggested that intervening lowland has been an effective barrier to dispersal in these species. We present a fine scale study which investigates historical patterning of *E. robertsi* across three mountaintops. These mountaintops in northern Queensland are located within an area <100 km^2^ and are the only known locations of *E. robertsi*. In a previous phylogeographic study, just twenty *E. robertsi* individuals were sampled. Small samples are likely to miss rare alleles and potentially under-represent the full phylogeographic history of a given locus. Limited distribution combined with anthropogenic disturbances (Coughran & Furse, 2010), have led to this species being categorised as critically endangered (Coughran & Furse, 2010).

*Temnosewellia albata* is the only known ectosymbiont associated with *E. robertsi*. Very little is known of its ecology, highlighting the need for further studies on *T. albata* to better understand the level of the association between this species and its host. In depth studies on another crayfish ectosymbiont, Branchiobdellida, have shown that associations previously regarded as mutualistic may actually be weakly parasitic in times of overabundance (Brown, Creed & Dobson, 2002; Brown et al., 2012). Although *T. albata* are not known to be parasitic we still expect that their life histories are closely aligned with their hosts. Currently, they are thought to be strictly host-specific and to undergo their entire life cycle on a single host (Jones & Lester, 1992; Sewell, Cannon & Blair, 2006). Infestations of *Temnosewellia* follow an over dispersion pattern, where a few hosts carry many individuals (Wild & Furse, 2004; C Hurry, pers. obs., 2014). For several species of *Temnosewellia* it has been shown that abundance, prevalence and flatworm body size are all positively correlated with crayfish size (*Euastacus sulcatus/Temnosewellia* spp. Wild & Furse, 2004; *Euastacus hystricosus/Temnosewellia batiola* C Hurry, 2014, unpublished data). These observations, offer a strong indication that newly hatched *Temnosewellia* may colonise small or young crayfish. As *Euastacus* carry their offspring for several months after hatching (McCormack, 2012), it is most likely that transmission of *Temnosewellia albata* is vertical. Even though *T. albata* are considered host-specific to *E. robertsi*, a single worm has been reported from a crayfish of the *Cherax depressus* complex sensu Riek, 1951, sampled at a site >500 km from the current habitat of *E. robertsi* (Sewell, Cannon & Blair, 2006). However, this identification has not been confirmed by further collection or molecular analyses.

Our study extends on previous phylogeographic research into *Euastacus robertsi* by using larger sample sizes and incorporating data from an additional locus. We then consider the phylogeography of an ectocommensal flatworm and seek to explore the longevity and history of the relationship between host and commensal. By conducting a comparative phylogeographic study we were able determine: (1) if there is evidence of past and present connectivity between populations of *Euastacus robertsi* and (2) if historical genetic patterns of colonisation and dispersal were congruent between *T. albata* and *E. robersti*. If *T. albata* shares a closely linked evolutionary history with its crayfish host, the topologies and relative depth of gene trees should be similar between the host and the flatworm.

## Material and Methods

### Study area

**Figure 1:**
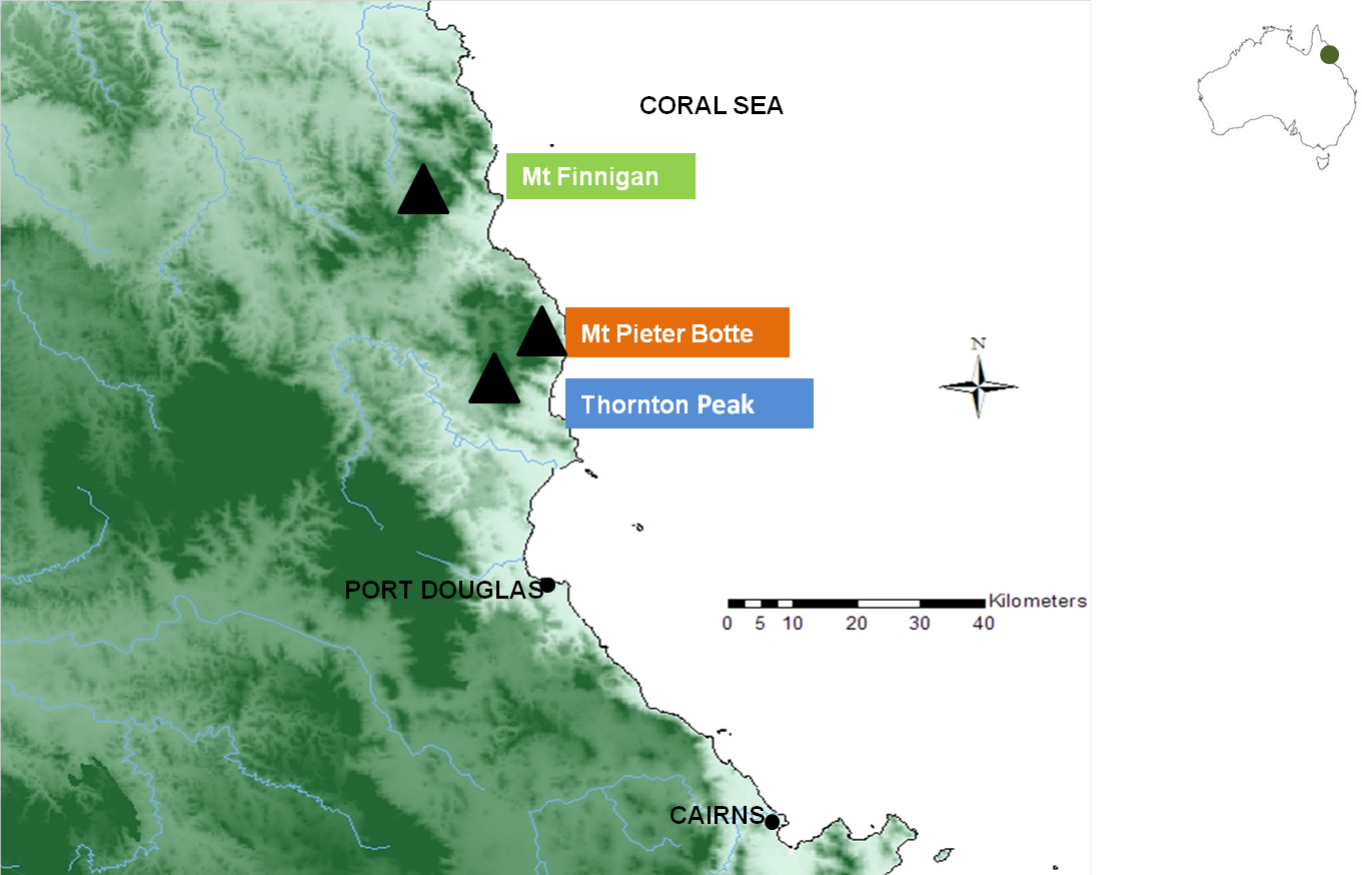
Map of north east Queensland, Australia which shows the three mountaintop habitats (▴) of *Euastacus robertsi* and *Temnosewellia albata*.

The study area was located in the Daintree rainforest, which is the largest continuous rainforest on the continent. Situated within the wet tropics in northern Queensland, Australia, the tropical climate has hot wet summers and cool dry winters. The three mountaintops inhabited by *T. albata* and *E. robersti* are Mount Pieter Botte (elevation 1,009 m), Thornton Peak (1,375 m) and Mount Finnigan (1,083 m) (Fig. 1, Table S1). The area which is <100 km^2^, is believed to contain the entire range of both species (Morgan, 1997; Ponniah & Hughes, 2006; Coughran & Furse, 2010). Samples were collected, as per Ponniah & Hughes (2004), in stream reaches at elevation >750 m. On each mountaintop one site was sampled except for Mount Finnigan where two stream reaches were sampled.

### DNA extraction, amplification, and sequencing

Total genomic DNA was extracted from the leg tissue of *E. robertsi* and from whole samples of *T. albata* as per methods outlined in Carini & Hughes (2006). A final edited 610 base pair fragment (*E. robertsi*) and 603 base pair fragment (*T. albata*) of the mtDNA COI gene was produced after polymerase chain reaction (PCR) using the COI primer set LCO-1490 and HCO-2198 of Folmer et al. (1994). PCR conditions were: denaturation of DNA occurred at 95 °C for 5 min, followed by 30 cycles of 94 °C denaturing for 1 min, 55 °C annealing for 30 s, and 72 °C extension for 1 min, followed by a final 68 °C extension step for 5 min. Dye terminator cycle sequencing reactions were used for sequencing (Perkin Elmer, Foster City, CA) as per manufacturer’s instructions. Sequencing was carried out on an Applied Biosystems (Foster City, CA) 3130xl automated sequencing machine.

We also wanted to include nuclear gene data in the analysis to compare phylogenetic patterns for the two species. Therefore, we included a 734 base pair edited fragment (*E. robertsi*) and a 692 base pair edited fragment (*T. albata*) of 28S ribosomal DNA. We used the primers Rd1a and Rd4b (Crandall, Harris & Fetzner, 2000). PCR conditions followed those of Crandall, Harris & Fetzner (2000).

The nucleotide sequences for COI and 28S were aligned and edited with SEQUENCHER v4.9 (Gene Codes Corporation). The mtDNA sequences were visually assessed for the occurrence of nuclear mitochondrial pseudogenes (numts) using techniques described in Bensasson et al. (2001) and none were found.

### Networks, phylogenetic trees and divergence estimates

For the 28S data we constructed haplotype networks using TCS v1.21 (Clement, Posada & Crandall, 2000) for *E. robertsi* and *T. albata*. Phylogenetic trees were constructed for the COI data using unique haplotypes. For the *E. robertsi* tree, *E. fleckeri* was selected as an out-group, as it has previously been shown to be the sister species of *E. robertsi* (Ponniah & Hughes, 2004). *Temnosewellia aphyodes* was chosen as an out-group for the *T. albata* tree, as it is the resident flatworm of *E*. *fleckeri* (Sewell, Cannon & Blair, 2006). We used jModeltest v0.1 (Posada, 2008) to choose the best-fit substitution model for each COI dataset. Using the Akaike information criterion the model selected for *T. albata* was TPM2uf + I + G and for *E. robertsi* was TIM3 + I. Tree construction for each data set was run using Bayesian analyses. MrBayes v3.1.2 (Huelsenbeck & Ronquist, 2001) was used for tree topology comparison, and BEAST v1.7.5 (Drummond & Rambaut, 2007; Drummond et al., 2012) was used to construct rooted ultrametric trees for comparison of node divergence times.

In MrBayes, a MCMC chain of 2,000,000 iterations was used with a sample frequency of 100. The first 25% of iterations were discarded as burn-in. MEGA v5.10 (Tamura et al., 2011) was used to calculate uncorrected percentage divergence between clades. In BEAST a lognormal relaxed clock model was first used to estimate divergence times of clades. However, the data could not reject a strict clock (ucld.stdev included zero); therefore, a strict clock model was used along with a coalescent constant size tree prior. Owing to lack of fossil calibration points and uncertainties in transferring molecular clock rates across taxa, we chose to incorporate a range of rates from the literature to place an approximate time-frame on COI divergences within the *E. robertsi* and *T. albata* datasets. Clock rates were used to describe a lognormal prior for the estimated clock rate, where 95% of the probability density was contained within highest and lowest values taken from the literature. For the temnocephalans these values were 0.0027 and 0.015 substitutions/site/lineage/million years (*Schmidtea mediterranea*, Platyhelminthes: Lazaro et al., 2011; *Dugesia*, Platyhelminthes: Sola et al., 2013). For the crayfish the values were 0.0083 and 0.012 substitutions/site/lineage/million years (*Chirocephalus*, Crustacea: Ketmaier, Argano & Caccone, 2003). Convergence, mixing and effective sample size of model parameters (>200) was assessed using the program Tracer v1.5 (Drummond & Rambaut, 2007) after running the analysis for 10^8^ generations.

To investigate the magnitude of genetic divergence within each taxon, without reliance on transformation using molecular clock rates, we fitted the COI and 28S datasets to a two population isolation-with-migration model (IM), implemented in the software IM (v.12/17/2009; Hey & Nielsen, 2004). Three pair-wise population comparisons (among the three mountaintop populations) were made for *E. robertsi* and *T. albata*. To ensure that results were consistent, each pair-wise comparison was run a minimum of three times (18 h/run) with different random number seeds. Model parameters of interest were taken from the peaks of the estimated distributions. These were population splitting time (*t*) scaled by the (unknown) geometric mean of the mutation rates for COI and 28S, and between-population migration rates (*m*1, *m*2).

## Results

### *Temnosewellia albata*

A total of 63 *T. albata* individuals were taken from 20 crayfish hosts sampled across five locations on three mountaintops (>700 m above sea level) (see Supplemental Information). Sixty one *T. albata* were sequenced for 603 bp of the COI mtDNA region. Seventeen unique haplotypes were identified (GenBank Accession: Table 1, see Supplemental Information). The target fragment contained no gaps and was variable at 91 sites (15%) of which 87 were parsimony informative (14%, Table 2). Between one and ten *T. albata* were sequenced for COI per crayfish (mean = 3.2). Average heterozygosity of *T. albata* sampled from one individual crayfish was not consistently lower compared to the average heterozygosity of *T. albata* sampled from a number of different crayfish. Due to sequencing issues, a small subset of the *T. albata* was used in the sequencing of the 28S ribosomal DNA region. For the 28S region, eight samples were sequenced for 692 base pairs and two unique haplotypes were identified (Table 1). The target fragment contained one gap at site 20 and had nine (1.3%) variable sites, all of which were parsimony informative (Table 2).

**Table 1:** GenBank accession numbers for *Temnosewellia* species. All sequences were generated as part of this study.

Species & molecular marker	Haplotype ID & GenBank accession number	Mountaintop location
*Temnosewellia albata* (COI)	TEM_FI1; KJ930397	Mt Finnigan/Thornton Peak
*Temnosewellia albata* (COI)	TEM_FI2; KJ930398	Mt Finnigan
*Temnosewellia albata* (COI)	TEM_FI3; KJ930399	Mt Finnigan
*Temnosewellia albata* (COI)	TEM_FI4; KJ930396	Mt Finnigan/Thornton Peak
*Temnosewellia albata* (COI)	TEM_FI5; KJ930400	Mt Finnigan
*Temnosewellia albata* (COI)	TEM_PB1; KJ930401	Mt Pieter Botte
*Temnosewellia albata* (COI)	TEM_PB2; KJ930402	Mt Pieter Botte
*Temnosewellia albata* (COI)	TEM_TP10 (D Blair); KJ930412	Mt Finnigan
*Temnosewellia albata* (COI)	TEM_TP7; KJ930409	Thornton Peak
*Temnosewellia albata* (COI)	TEM_TP2; KJ930404	Thornton Peak
*Temnosewellia albata* (COI)	TEM_TP3; KJ930405	Thornton Peak
*Temnosewellia albata* (COI)	TEM_TP8; KJ930410	Thornton Peak
*Temnosewellia albata* (COI)	TEM_TP9; KJ930411	Thornton Peak
*Temnosewellia albata* (COI)	TEM_TP4; KJ930406	Thornton Peak
*Temnosewellia albata* (COI)	TEM_TP5; KJ930407	Thornton Peak
*Temnosewellia albata* (COI)	TEM_TP6; KJ930408	Thornton Peak
*Temnosewellia albata* (COI)	TEM_TP1; KJ930403	Thornton Peak
*Temnosewellia aphyodes* (COI)	530FR (D.Blair); KJ958928	Mt Lewis
*Temnosewellia albata* (28S)	TEM_1; KJ941013	Mt Finnigan/Thornton Peak
*Temnosewellia albata* (28S)	TEM_2; KJ941014	Mt Pieter Botte/Thornton Peak

**Table 2:** Comparison of genetic diversity between *Temnosewellia albata* and *Euastacus robertsi*.

Species name and molecular marker	*n*	Haplotypes	*Hd*	*π*	*S*
*Temnosewellia albata* COI	61	17	0.89	0.071	91
*Temnosewellia albata* 28S	8	2	0.57	0.0096	9
*Euastacus robertsi* COI	64	6	0.65	0.023	31
*Euastacus robertsi* 28S	24	4	0.67	0.0076	12

**Notes.**

*Hd* haplotype diversity *π* nucleotide diversity *S* segregating sites

### *Euastacus robertsi*

For *E. robertsi*, 610 base pairs of the COI region were available for 64 individuals (including 16 from Genbank) (see Supplemental Information). Six unique haplotypes were discovered (GenBank accession: Table 3, see Supplemental Information). The target fragment contained no gaps and was variable at 31 sites (5%) of which 30 sites (97%) were parsimony informative (Table 2). The out-group consisted of three sequences from *E. fleckeri* (including two from GenBank). Twenty five of the *E. robertsi* individuals were sequenced for 733 base pairs of 28S ribosomal DNA and, four unique haplotypes were identified (Table 3). The target fragment contained no gaps and had 12 variable sites (1.6%; Table 2) 11 sites were parsimony informative (92%).

### Genetic variation

#### Comparison of COI tree topology between species

**Figure 2:**
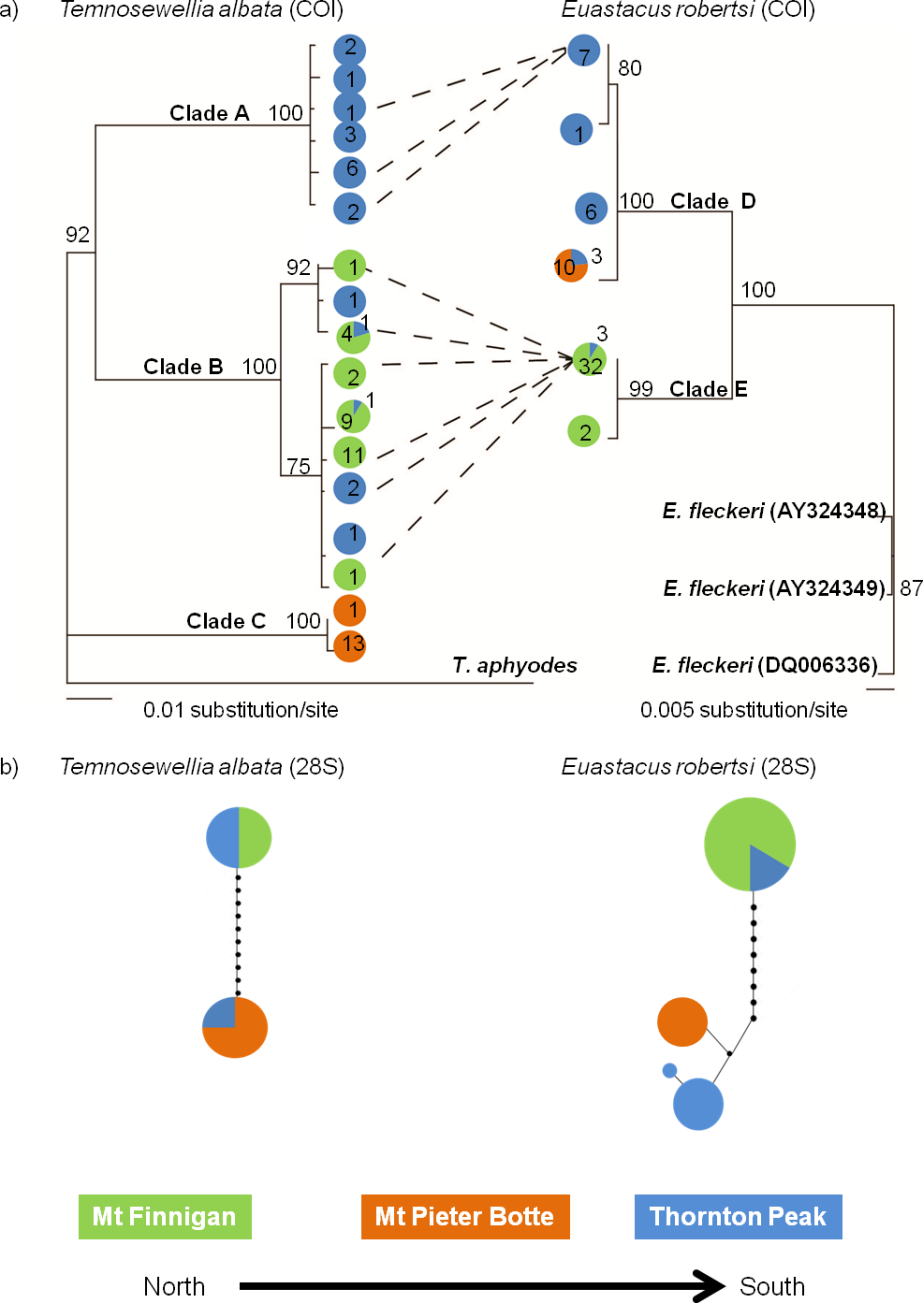
Comparison of two Bayesian (BA) consensus topologies of the COI mtDNA datasets (A), and a parsimony network generated on TCS, of 28S ribosomal DNA sequence data (B). (A) BA posterior probabilities are shown above the node. The colours represent the location where the haplotype was sampled. Numbers represent the number of individuals sampled with that haplotype. Dashed lines represent a specific linkage where flatworms were sampled from hosts with that haplotype. (B) Haplotype frequency is indicated by the circle size (smallest 1, largest 8). The circle fill colour indicates sample site. Circles on connecting lines indicate the number of base pair mutations between haplotypes.

Tree topologies for both taxa featured two or three deeply divided in-group clades (Fig. 2A). The main difference between taxa was, for *T. albata* there was division into three well-supported clades (clades A, B, C), whereas for their crayfish hosts *E. robertsi*, two well-supported clades (D, E) were identified. The three *T. albata* clades corresponded strongly with the three separate mountaintop populations, although one clade (B) included a mixture of samples from Thornton Peak and Mt Finnigan. The spatial pattern of the two clades in the *E. robertsi* tree grouped together samples from Mt Pieter Botte and Thornton Peak together (clade D). Clade E was comprised mostly of samples from Mt Finnigan.

#### Comparison of 28S tree topology between species

The 28S haplotype networks were similar in structure for both species, as both feature two groups of haplotypes separated by quite a large mutational distance (10–11 bases; Fig. 2B). In both networks, there were samples collected at Thornton Peak (blue: Fig. 2B) which shared the same haplotype, or a very similar haplotype (one or two bases different), to a number of the samples collected at Mt Pieter Botte. Also, for both species, haplotype sharing was evident for samples collected at Thornton Peak and Mount Finnigan. Another point of congruence between the species is that there was a large mutational distance separating Mt Pieter Botte and some samples from Mt Finnigan. Finally, the 28S haplotype networks showed strong similarities to the COI phylogenetic trees; the main difference being that the *T. albata* 28S data exhibited two clades compared to three for the COI data.

#### Divergence estimates and isolation-with-migration model

**Figure 3:**
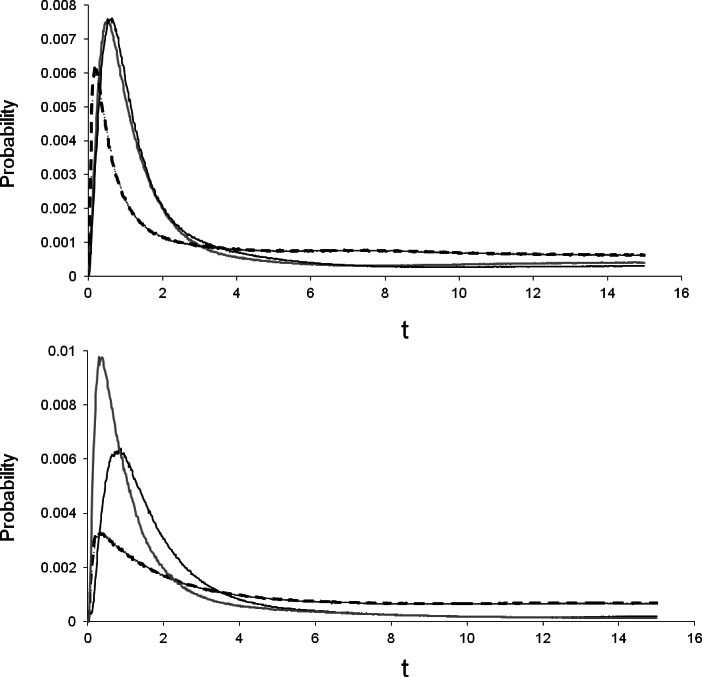
Isolation by migration model, using COI and 28S, showing the pairwise difference in divergence between three mountains. Black line, Thornton peak & Mt Finnigan; grey line, Thornton peak & Mt Pieter Botte; dashed line, Mt Finnigan & Mt Pieter Botte.

Percent divergence of the *T. albata* COI clades (shown in Fig. 2) was 12% for both clades A–C and B–C, and 10% for clades A–B. The median divergence time calculated by BEAST for clades A–B was ∼11 mya (Table 4). Percent divergence for *E. robertsi* for clades D–E was 5%. The median divergence time calculated by BEAST for clades D–E was ∼2.6 mya. Evaluation of the population divergence time parameter (*t*), incorporating both COI and 28S data in an IM model revealed no difference between *T. albata* and *E. robertsi* for the three among-mountaintop comparisons (Fig. 3; Table 5). Mean point estimates of *t* were 0.303 for crayfish and 0.338 for temnocephalans with broadly overlapping 95% credibility intervals. Note that these values are unscaled parameter estimates. Conversion into units of real time would require scaling these values by the (unknown) substitution rates for each locus and by the (unknown) generation time of each species.

**Table 3:** GenBank accession numbers for *Euastacus robertsi*. All the new sequences that were generated as part of this study are highlighted in boldface type.

Molecular marker	Haplotype ID & GenBank accession number	Mountain top location
COI	FI-A; DQ006368, DQ006372, DQ006377,	Mt Finnigan/Thornton Peak
	DQ006378, AY800362, DQ006369, AY324346	
COI	FI-P; KJ939254	Mt Finnigan
COI	TP1; DQ006370, DQ006376	Thornton Peak
COI	PB1; DQ006373, AY800364, AY324347	Mt Pieter Botte
COI	TP3; AY800363	Thornton Peak
COI	TP2; KJ939253	Thornton Peak
28S	F1; EU920988	Mt Finnigan/Thornton Peak
28S	P1; KJ941016	Mt Pieter Botte
28S	T1; KJ941015	Thornton Peak
28S	T2; KJ941017	Thornton Peak

**Table 4:** Divergence estimates between clades (see Fig. 2A). Comparison is between sequenced COI haplotypes, for *Temnosewellia albata* and *Euastacus robertsi* in Queensland, Australia.

	Pairwise comparison	Substitutions/site/lineage/million years		Diverged (mya)	±95% HPD (mya)
		Lower	Upper		
*Temnosewellia albata*	Clade A–B	0.0027	0.015	11	3.4–25
	Clade A–B–C	0.0027	0.015	15	4.4–32
*Euastacus robertsi*	Clade D–E	0.0083	0.012	2.6	1.5–4.3

**Table 5:** Parameter estimates from an isolation with migration model for three pairwise mountaintop population comparison.

	*Temnosewellia albata*				*Euastacus robertsi*			
Comparison	Parameters	HiPt	HPD90Lo	HPD90Hi	Parameters	HiPt	HPD90Lo	HPD90Hi
	qF	1.702	0.361	6.448	qF	0.1700	0.0154	1.126
1. Mt Finnigan	qP	0.147	0.049	1.522	qP	0.0163	0.0163	0.992
–	qA	44.82	19.75	103.1	qA	30.85	6.423	32.50
2. Mt Pieter Botte	t	0.308	0.053?	14.99?	t	0.173	0.022?	12.69?
	mF	0.005	0.005	0.515	mF	0.005	0.005	0.995
	mP	0.005	0.005	1.285	mP	0.005	0.005	1.355
	qF	0.965	0.170	4.596	qF	0.1	0.02	0.86
1. Mt Finnigan	qT	3.440	0.313	41.802	qT	1.661	0.2518	7.100
–	qA	38.46	13.03?	103.1	qA	27.545	10.52?	91.49?
2. Thornton Peak	t	0.878	0.068?	7.238?	t	0.6225	0.0375?	14.99?
	mF	0.005	0.005?	8.325?	mF	0.005	0.0050?	5.755?
	mT	1.225	0.005	7.27	mT	0.275	0.015	3.855
	qT	3.563	0.6600	16.75	qT	3.020	0.3583	16.63
1. Thornton Peak	qP	0.087	0.087	1.478	qP	0.0217	0.0217	0.5852
–	qA	49.21	13.03?	171.6?	qA	23.18	8.446	89.94
2. Mt Pieter Botte	t	0.292	0.068?	7.328?	t	0.5175	0.0225?	14.99?
	mT	0.055	0.005	1.685	mT	0.265	0.005	3.565
	mP	0.005	0.0050?	5.845	mP	0.005	0.0050?	8.795?

**Notes.**

**HiPt** the value of the bin with the highest count **HPD90Lo** lower bound of the estimated 90% highest posterior density (HPD) interval. A question mark ‘?’ indicates unreliable or limit due to flat or incomplete posterior probability distribution sampled **HPD90Hi** upper bound of the estimated 90% highest posterior density (HPD) interval **q** the effective population size, population indicated by the letter (F, T, P) qA ancestral population **m** the migration rate per gene copy per generation, letters indicate the population (F, T, P) **t** a divergence estimate (not transformed to years)

Assessment of among-mountaintop migration using the IM model indicated no migration was compatible with the data for most pairwise comparisons (Table 5). However, the data did support non-zero migration from the Mt Finnigan population into the Thornton Peak population for both taxa: crayfish (*m_FtoT_* = 0.2; i.e., parameter estimate converted to demographic units representing effective number of migrants per generation), and temnocephalans (*m_FtoT_* = 1.9). Non-zero migration was also detected for crayfish from the Mt Pieter Botte population moving into the Thornton Peak population (Table 5). However, the temnocephalan data did not mirror this pattern.

## Discussion

By studying molecular data of a critically endangered freshwater crayfish and its ectocommensal flatworm, we found evidence that the phylogeographic patterning in *T. albata* is consistent with that of the host, *E. robertsi*. We suggest that populations on the mountain peaks separated sometime during the Pliocene. Contrary to earlier research on *E. robersti* (Ponniah & Hughes, 2006) we found some haplotype sharing between these mountains. We suggest that haplotype sharing among mountaintops for both species is a product of post-divergence gene flow, although dispersal events between these mountain peaks have been infrequent.

The Greater Daintree National Park, a vast area of land which includes the mountains in this study, is considered to be more than 135 million years old and is a hotspot of biodiversity (Hopkins et al., 1996). Throughout the Tertiary period rainfall remained at levels high enough to sustain extensive rainforest (Frakes, 1999), with a shift during the late tertiary to drier fire-prone sclerophyll forest (Truswell, 1993). These conditions during the Pliocene are believed to have had a significant impact on population distribution and structure of fauna and flora in the north and the coastal east of Australia (Schneider, Cunningham & Moritz, 1998; Schneider & Moritz, 1999). Rainforest contractions occurred in the wet tropics during Pleistocene glacial periods (Kershaw, 1994). Over the last 230,000 years rainforest expansions have occurred during wetter interglacial periods before being replaced by drier rainforest and sclerophyll vegetation in drier glacial periods (Kershaw & van der Kaars, 2007). These more recent periods of rainforest expansion may have facilitated movement among mountaintop populations and produced the observed pattern of unidirectional migration inferred in the genetic data of both species.

### Shared phylogeographic patterning

At two independent loci we identified haplotype sharing between mountains for the flatworm and its host. We cannot say with absolute certainty if these shared haplotypes are the result of gene flow or retention of ancestral haplotypes, but analysis using the IM model was compatible with low levels of unidirectional gene flow after population isolation. The pattern shared by crayfish and flatworms was for migration from Mt Finnigan into Thornton Peak. We established that, in both datasets, Mt Finnigan and Mt Pieter Botte were isolated from each other due to a lack of haplotype sharing with no evidence of migration. It has long been postulated that intervening lowlands are effective barriers to dispersal for Queensland *Euastacus* (Ponniah & Hughes, 2004; Ponniah & Hughes, 2006). It is also likely that the lack of migration for this species could be attributed to these two mountains being on completely separate ridges. Furthermore, we consider it possible that Mt Finnigan and Thornton Peak may have once shared a ridge making historical connections between them more likely. These connections may have been present either overland or through historical stream connections. Historical connections between these two mountains have been found for the beetle *Philipis* (Baehr, 1995). As *Euastacus* are known to be able to survive for long periods out of water they have the ability to traverse over land (Furse & Wild, 2002), although overland dispersal may be rarer in some species. Intervening high points along ridge lines may have allowed for historical migration pathways. Current elevations between these mountains are no lower than 350 m at some places. Therefore, although it has been shown to be a rare occurrence, it is possible that migration between sites is possible, at least between two of the mountain ridges.

The congruence that we observed in the phylogeographic pattern of *T*. *albata* and its crayfish host suggests that their evolutionary histories are spatially linked; therefore, if hosts are capable of overland dispersal, so are the flatworms. The exact mechanism of dispersal for *Temnosewellia* is unknown. As the genus is generally considered to be host-specific it is expected that, like other temnocephalans, they undergo their entire life cycle and subsequent generations on a single host crayfish (Sewell, Cannon & Blair, 2006). The mechanisms that allow them to survive the moult phase of their hosts are not known; however, observations by Haswell (1983) and Nichols (1975), on closely related temnocephalans, noted that they may be able to survive for some time in the absence of a host. Even though the flatworm’s ectoderm is somewhat prone to desiccation (Haswell, 1909; C Hurry, pers. obs., 2014), flatworms may still be able to disperse overland with their host due to the durability of their unhatched eggs. *Temnosewellia* will lay tens to hundreds of eggs which stick firmly to the exoskeleton of the crayfish (Wild & Furse, 2004; C Hurry, pers. obs., 2014). Eggs are enclosed in a tough outer coating and have a large fluid filled cavity (Haswell, 1909), which may prevent desiccation of the unhatched young allowing long distance movement in the absence of water. As so little is known of *Temnosewellia* this hypothesis has not been tested and further work is needed to determine dispersal mechanisms in these flatworms.

### Time of divergence

As low levels of migration were detected it should be easier to detect founding events. Our results show that, for both these species, isolation and divergence among refugial mountaintop populations was old enough to have resulted in accumulation of mutational differences. Comparison of COI divergence times for a node marking the split between Mt Finnigan and Thornton Peak for both taxa suggested the temnocephalan divergence may be older than the crayfish (∼11 mya compared to ∼2.6 mya). However this result is contingent on calibration using molecular clock rates from the literature. Numerous studies have highlighted variability in substitution rates between taxa (Wilke, Schultheiß & Albrecht, 2009; Lanfear, Welch & Bromham, 2010), so caution is required in interpreting the divergence times presented here. By taking a different population-based approach—using the multilocus, isolation-with-migration model—we showed that the population splitting parameter *t* was indistinguishable between the two species. This comparison incorporates data from another locus in addition to COI, and does not depend on application of molecular clock rates (which may not be appropriate for our study species). However it does have the drawback of not being expressed in units of absolute time. Weighing-up both of these results leads us to conclude that either (1) the mountaintop divergences of both species did occur contemporaneously, but that a greater number of substitutions have become fixed in the mitochondrial genome of the flatworm compared to the crayfish (i.e., their divergence rates are different) or (2) *T. albata* did diverge earlier than their hosts. The second assumption is entirely possible if in the past *T. albata* had a different host which has now become extinct. A different host could either be an ancestral *Euastacus* which was not sampled as part of this study or another crustacean host altogether. If we were able to confirm the authenticity of the single *T. albata* sampled upon the crayfish *Cherax depressus* ∼515 km south of Mount Pieter Botte (Sewell, Cannon & Blair, 2006); we may find that in the past the distribution of this species was much wider. However, as this hypothesis is based upon just one sample we are cautious in offering this interpretation. Our calculations support a separation sometime during the Pliocene. As previously stated, conditions during the Pliocene are believed to have had a significant impact on population distribution and structure of fauna and flora in the north and the coastal east of Australia due to vicariance events.

Due to the close association that *Temnosewellia* share with their host they may be, in future studies, considered to be a suitable proxy in resolving phylogeographic patterning in their hosts. Nieberding & Olivieri (2007) tell us that ‘parasites’ that act as suitable proxy species are without intermediate hosts and have no phase of living independently of their host. Equally they are individuals which display smaller *N_e_* at the population level and exhibit lower gene flow than their hosts among populations. These factors combined allow them to display a stronger population structure than their host, making them especially useful in cases where hosts are rare or hard to sample in large numbers. As *Temnosewellia* satisfies many of these criteria their role in future studies may be to help resolve host phylogenies or phylogeographic history. The existence in this study of three deeply divided COI lineages in the temnocephalan compared with two in the crayfish may indeed indicate that the ectocommensal genealogy records part of the crayfish history that is lost due to stochastic sorting of lineages and extinction/recolonisation events. One possibility is that a third crayfish mtDNA lineage did exist on Mt Pieter Botte, but was replaced by the Thornton Peak mtDNA lineage following a colonisation event. In this scenario the ectocommensal history acts as proxy for the crayfish history. However the extra temnocephalan mtDNA lineage might also be explained by lineage retention or by failure to detect a corresponding third lineage in our crayfish sample.

We were able to demonstrate that the association between *E. robertsi* and *T. albata* has likely persisted over several million years. The results from our study are applicable to host-commensal relationships worldwide, as they show that shared histories between such close commensal species may span millions of years. A growing number of examples in the literature are demonstrating that symbionts can be used to infer host history for conservation gains (Colwell, Dunn & Harris, 2012), which highlight the importance of studying symbiotic species alongside their hosts. We suggest that future phylogeographic studies exploit host-commensal interactions to provide objective measures of biodiversity, population subdivisions and phylogeographic information in the host. These interactions should be considered in management plans for crayfish species, especially as this technique may prove useful when host numbers are small, due to rarity or low catch rates.

## Supplemental Information

10.7717/peerj.552/supp-1 Table S1 Sample locations for *Temnosewellia albata* and *Euastacus robertsi* *Temnosewellia albata* (T.a); *Euastacus robertsi* (E.r).

10.7717/peerj.552/supp-2 Table S2 *Euastacus robersti*-details of COI and 28S sequences used in phylogenetic analysis ^**1**^ Ponniah & Hughes 2006
^2^ Shull et al., 2005
^3^ Hurry et al. 2014 (current publication)
^4^ Toon et al. 2009.

10.7717/peerj.552/supp-3 Table S3 *Temnosewellia albata*—Details of COI and 28S sequences used in phylogenetic analysis ^1^ Hurry et al. 2014 (current publication)
*-not sequenced.

## Additional Information and Declarations

### Field Study Permissions

The following information was supplied relating to field study approvals (i.e., approving body and any reference numbers): The Queensland National Parks and Wildlife Service provided the necessary permits to collect samples as part of an earlier project by Mark Ponniah, see references 2004 & 2006.
