# Supplementary material for: Shared phylogeographic patterns between the ectocommensal flatworm Temnosewellia albata and its host, the endangered freshwater crayfish Euastacus robertsi
Source: PeerJ. 2014 Sep 25;2:e552. doi: 10.7717/peerj.552 (PMC4179389; doi:10.7717/peerj.552)
Supplement: Table S1 — Temnosewellia albata (T.a); Euastacus robertsi (E.r). [file peerj-02-552-s001.docx]

| **Stream name** | **Location** | **Latitude** | **Longitude** | **Total E.r** | **Total T.a** |
| --- | --- | --- | --- | --- | --- |
| Annan Creek | Mt Finnigan | -15°49’27” | 145°16’30” | 20 | 19 from 6 E.r |
| Parrots Creek | Mt Finnigan | -15°49’00” | 145°16’00” | 14 | 8 from 3 E.r |
| Horan’s Creek | Mt Finnigan | -15°49’12” | 145°16’11” | 1 | 1 from 1 E.r |
| Roaring Meg River | Mt Pieter Botte | -16°03’59” | 145°25’10” | 14 | 14 from 3 E.r |
| Hilda Creek | Thornton Peak | -16°09’43” | 145°22’00” | 23 | 21 from 7 E.r |
