# Supplementary material for: Shared phylogeographic patterns between the ectocommensal flatworm Temnosewellia albata and its host, the endangered freshwater crayfish Euastacus robertsi
Source: PeerJ. 2014 Sep 25;2:e552. doi: 10.7717/peerj.552 (PMC4179389; doi:10.7717/peerj.552)
Supplement: Table S2 — 1 Ponniah & Hughes 2006 2 Shull et al., 2005 3 Hurry et al. 2014 (current publication) 4 Toon et al. 2009. [file peerj-02-552-s002.docx]

| \| **Identification** \| **Mountain** \| **Stream** \| **Haplotype ID & Genbank accession #** \| \| \| --- \| --- \| --- \| --- \| --- \| \|  \|  \|  \| **CO1** \| **28S** \| \| clone robTH \| Thornton Peak \| Hilda Creek \| AY324347**^1^** \|  \| \| Euastacus fleckeri-clone fleckML \| OUTGROUP- Mt Lewis \| Leichhardt Ck \| AY324348**^1^** \|  \| \| Euastacus fleckeri-clone fleckMS \| OUTGROUP \| Unknown \| AY324349**^1^** \|  \| \| Euastacus fleckeri-KC2668 \| OUTGROUP- Mt Lewis \| Leichhardt Ck \| DQ006336**^1^** \|  \| \| FI1 \| Mt Finnigan \| Annan Ck \| FI-A; DQ006368**^2^** \|  \| \| FI10 \| Mt Finnigan \| Annan Ck \| FI-A; DQ006368**^2^** \| F1; EU920988**^4^** \| \| FI10J \| Mt Finnigan \| Annan Ck \| FI-A; DQ006368**^2^** \|  \| \| FI11 \| Mt Finnigan \| Annan Ck \| FI-A; DQ006368**^2^** \| F1; EU920988**^4^** \| \| FI11j \| Mt Finnigan \| Annan Ck \| FI-A; DQ006368**^2^** \|  \| \| FI12 \| Mt Finnigan \| Annan Ck \| FI-A; DQ006368**^2^** \|  \| \| FI13 \| Mt Finnigan \| Annan Ck \| FI-A; DQ006368**^2^** \|  \| \| FI13J \| Mt Finnigan \| Parrot Ck \| FI-P; KJ939254**^3^** \|  \| \| FI14 \| Mt Finnigan \| Annan Ck \| FI-A; DQ006368**^2^** \| F1; EU920988**^4^** \| \| FI14J \| Mt Finnigan \| Parrot Ck \| FI-A; DQ006368**^2^** \|  \| \| FI15 \| Mt Finnigan \| Parrot Ck \| FI-A; DQ006368**^2^** \|  \| \| FI16 \| Mt Finnigan \| Parrot Ck \| FI-A; DQ006368**^2^** \| F1; EU920988**^4^** \| \| FI17 \| Mt Finnigan \| Parrot Ck \| FI-A; DQ006368**^2^** \| F1; EU920988**^4^** \| \| FI18 \| Mt Finnigan \| Parrot Ck \| FI-A; DQ006368**^2^** \| F1; EU920988**^4^** \| \| FI19 \| Mt Finnigan \| Annan Ck \| FI-A; DQ006368**^2^** \| F1; EU920988**^4^** \| \| FI1j \| Mt Finnigan \| Parrot Ck \| FI-A; DQ006368**^2^** \|  \| \| FI2 \| Mt Finnigan \| Annan Ck \| FI-A; DQ006368**^2^** \|  \| \| FI2J \| Mt Finnigan \| Annan Ck \| FI-A; DQ006368**^2^** \|  \| \| FI3 \| Mt Finnigan \| Annan Ck \| FI-A; DQ006368**^2^** \|  \| \| FI3J \| Mt Finnigan \| Parrot Ck \| FI-P; KJ939254**^3^** \|  \| \| FI4 \| Mt Finnigan \| Annan Ck \| FI-A; DQ006368**^2^** \| F1; EU920988**^4^** \| \| FI5 \| Mt Finnigan \| Annan Ck \| FI-A; DQ006368**^2^** \|  \| \| FI5J \| Mt Finnigan \| Parrot Ck \| FI-A; DQ006368**^2^** \|  \| \| FI6 \| Mt Finnigan \| Annan Ck \| FI-A; DQ006368**^2^** \| F1; EU920988**^4^** \| \| FI7 \| Mt Finnigan \| Annan Ck \| FI-A; DQ006368**^2^** \|  \| \| FI7J \| Mt Finnigan \| Parrot Ck \| FI-A; DQ006368**^2^** \|  \| \| FI8 \| Mt Finnigan \| Annan Ck \| FI-A; DQ006368**^2^** \| F1; EU920988**^4^** \| \| KC2669 \| Mt Finnigan \| Parrot Ck \| FI-A; DQ006368**^2^** \|  \| \| KC2670 \| Mt Finnigan \| Horan’s Ck \| FI-A; DQ006369**^2^** \|  \| \| KC2674 \| Thornton Peak \| Hilda Creek \| TP1; DQ006370**^2^** \|  \| \| KC2737 \| Mt Finnigan \| Parrot Ck \| FI-A; DQ006371**^2^** \|  \| \| KC2738 \| Mt Finnigan \| Parrot Ck \| FI-A; DQ006372**^2^** \|  \| \| KC2776 \| Mt Pieter Botte \| R.Meg River \| PB1; DQ006373**^2^** \|  \| \| KC2777 \| Mt Pieter Botte \| R.Meg River \| PB1; DQ006374**^2^** \|  \| \| KC2778 \| Thornton Peak \| Hilda Creek \| TP1; DQ006375**^2^** \|  \| \| KC2779 \| Thornton Peak \| Hilda Creek \| TP1; DQ006376**^2^** \|  \| \| KC2780 \| Mt Finnigan \| Annan Ck \| FI-A; DQ006377**^2^** \|  \| \| KC2781 \| Mt Finnigan \| Annan Ck \| FI-A; DQ006378**^2^** \|  \| \| PB2 \| Mt Pieter Botte \| R.Meg River \| PB1; DQ006373**^2^** \|  \| \| PB1 \| Mt Pieter Botte \| R.Meg River \| PB1; DQ006373**^2^** \|  \| \| PB3 \| Mt Pieter Botte \| R.Meg River \| PB1; DQ006373**^3^** \|  \| \| PB37 \| Mt Pieter Botte \| R.Meg River \|  \| P1; KJ941016**^3^** \| \| PB38 \| Mt Pieter Botte \| R.Meg River \|  \| P1; KJ941016**^3^** \| \| PB39 \| Mt Pieter Botte \| R.Meg River \|  \| P1; KJ941016**^3^** \| \| PB4 \| Mt Pieter Botte \| R.Meg River \| PB1; DQ006373**^2^** \|  \| \| PB40 \| Mt Pieter Botte \| R.Meg River \|  \| P1; KJ941016**^3^** \| \| PB41 \| Mt Pieter Botte \| R.Meg River \|  \| P1; KJ941016**^3^** \| \| PB5 \| Mt Pieter Botte \| R.Meg River \| PB1; DQ006373**^2^** \|  \| \| PB6 \| Mt Pieter Botte \| R.Meg River \| PB1; DQ006373**^2^** \|  \| \| PB7 \| Mt Pieter Botte \| R.Meg River \| PB1; DQ006373**^2^** \|  \| \| RA cytochrome \| Mt Finnigan \| Annan Creek \| FI-A; AY800362**^1^** \|  \| \| RB cytochrome \| Thornton Peak \| Hilda Ck \| TP3; AY800363**^1^** \|  \| \| RC cytochrome \| Mt Pieter Botte \| R.Meg River \| PB1; AY800364**^1^** \|  \| \| robFIN \| Mt Finnigan \| Annan Creek \| FI-A; AY324346**^1^** \|  \| \| TP1 \| Thornton Peak \| Hilda Ck \| TP1; DQ006370**^2^** \|  \| \| TP2 \| Thornton Peak \| Hilda Ck \| TP1; DQ006370**^2^** \|  \| \| TP25 \| Thornton Peak \| Hilda Ck \|  \| T1; KJ941015**^3^** \| \| TP28 \| Thornton Peak \| Hilda Ck \| TP1; DQ006370**^2^** \| T1; KJ941015**^3^** \| \| TP29 \| Thornton Peak \| Hilda Ck \|  \| T1; KJ941015**^3^** \| \| TP3 \| Thornton Peak \| Hilda Ck \| TP1; DQ006370**^2^** \|  \| \| TP31 \| Thornton Peak \| Hilda Ck \|  \| T1; KJ941015**^3^** \| \| TP30 \| Thornton Peak \| Hilda Ck \| TP2; KJ939253**^3^** \| T1; KJ941015**^3^** \| \| TP32 \| Thornton Peak \| Hilda Ck \| FI-A; DQ006368**^2^** \| T2; KJ941017**^3^** \| \| TP33 \| Thornton Peak \| Hilda Ck \| FI-A; DQ006368**^2^** \| F1; EU920988**^4^** \| \| TP34 \| Thornton Peak \| Hilda Ck \| FI-A; DQ006368**^2^** \| F1; EU920988**^4^** \| \| TP36 \| Thornton Peak \| Hilda Ck \|  \| T1; KJ941015**^3^** \| \| TP4 \| Thornton Peak \| Hilda Ck \| PB1; DQ006373**^2^** \|  \| \| TP5 \| Thornton Peak \| Hilda Ck \| TP3; AY800363**^1^** \|  \| \| TP6 \| Thornton Peak \| Hilda Ck \| TP3; AY800363**^1^** \|  \| \| TP7 \| Thornton Peak \| Hilda Ck \| TP3; AY800363**^1^** \|  \| \| TP8 \| Thornton Peak \| Hilda Ck \| TP3; AY800363**^1^** \|  \| \| TP9 \| Thornton Peak \| Hilda Ck \| TP3; AY800363**^1^** \|  \| |
| --- | --- | --- | --- | --- | --- | --- | --- | --- | --- | --- | --- | --- | --- | --- | --- | --- | --- | --- | --- | --- | --- | --- | --- | --- | --- | --- | --- | --- | --- | --- | --- | --- | --- | --- | --- | --- | --- | --- | --- | --- | --- | --- | --- | --- | --- | --- | --- | --- | --- | --- | --- | --- | --- | --- | --- | --- | --- | --- | --- | --- | --- | --- | --- | --- | --- | --- | --- | --- | --- | --- | --- | --- | --- | --- | --- | --- | --- | --- | --- | --- | --- | --- | --- | --- | --- | --- | --- | --- | --- | --- | --- | --- | --- | --- | --- | --- | --- | --- | --- | --- | --- | --- | --- | --- | --- | --- | --- | --- | --- | --- | --- | --- | --- | --- | --- | --- | --- | --- | --- | --- | --- | --- | --- | --- | --- | --- | --- | --- | --- | --- | --- | --- | --- | --- | --- | --- | --- | --- | --- | --- | --- | --- | --- | --- | --- | --- | --- | --- | --- | --- | --- | --- | --- | --- | --- | --- | --- | --- | --- | --- | --- | --- | --- | --- | --- | --- | --- | --- | --- | --- | --- | --- | --- | --- | --- | --- | --- | --- | --- | --- | --- | --- | --- | --- | --- | --- | --- | --- | --- | --- | --- | --- | --- | --- | --- | --- | --- | --- | --- | --- | --- | --- | --- | --- | --- | --- | --- | --- | --- | --- | --- | --- | --- | --- | --- | --- | --- | --- | --- | --- | --- | --- | --- | --- | --- | --- | --- | --- | --- | --- | --- | --- | --- | --- | --- | --- | --- | --- | --- | --- | --- | --- | --- | --- | --- | --- | --- | --- | --- | --- | --- | --- | --- | --- | --- | --- | --- | --- | --- | --- | --- | --- | --- | --- | --- | --- | --- | --- | --- | --- | --- | --- | --- | --- | --- | --- | --- | --- | --- | --- | --- | --- | --- | --- | --- | --- | --- | --- | --- | --- | --- | --- | --- | --- | --- | --- | --- | --- | --- | --- | --- | --- | --- | --- | --- | --- | --- | --- | --- | --- | --- | --- | --- | --- | --- | --- | --- | --- | --- | --- | --- | --- | --- | --- | --- | --- | --- | --- | --- | --- | --- | --- | --- | --- | --- | --- | --- | --- | --- | --- | --- | --- | --- | --- | --- | --- | --- | --- | --- | --- | --- | --- | --- | --- | --- | --- | --- | --- | --- | --- | --- | --- | --- | --- | --- | --- | --- | --- | --- | --- | --- | --- | --- | --- | --- | --- | --- | --- | --- | --- | --- | --- | --- | --- | --- | --- | --- | --- | --- | --- |
