# Supplementary material for: Shared phylogeographic patterns between the ectocommensal flatworm Temnosewellia albata and its host, the endangered freshwater crayfish Euastacus robertsi
Source: PeerJ. 2014 Sep 25;2:e552. doi: 10.7717/peerj.552 (PMC4179389; doi:10.7717/peerj.552)
Supplement: Table S3 — 1 Hurry et al. 2014 (current publication) *-not sequenced. [file peerj-02-552-s003.docx]

| **Identification** | **Mountain** | **Stream** | **Haplotype ID and Genbank accession #^1^** | | **Corresponding Euastacus robersti** |
| --- | --- | --- | --- | --- | --- |
|  |  |  | **CO1** | **28s** |  |
| FI12.1 | Mt Finnigan | Annan Ck |  | TEM_1; KJ941013 | FI12 |
| FI12.2 | Mt Finnigan | Annan Ck | TEM_FI1; KJ930397 |  | FI12 |
| FI12.4 | Mt Finnigan | Annan Ck | TEM_FI1; KJ930397 |  | FI12 |
| FI12.5 | Mt Finnigan | Annan Ck | TEM_FI1; KJ930397 |  | FI12 |
| FI14.1 | Mt Finnigan | Annan Ck | TEM_FI1; KJ930397 |  | FI14 |
| FI14.2 | Mt Finnigan | Annan Ck | TEM_FI2; KJ930398 |  | FI14 |
| FI14.3 | Mt Finnigan | Annan Ck | TEM_FI3; KJ930399 |  | FI14 |
| FI16.1 | Mt Finnigan | Parrots Ck | TEM_FI4; KJ930396 |  | FI16 |
| FI16.2 | Mt Finnigan | Parrots Ck | TEM_FI1; KJ930397 |  | FI16 |
| FI16.3 | Mt Finnigan | Parrots Ck | TEM_FI4; KJ930396 |  | FI16 |
| FI17.1 | Mt Finnigan | Parrots Ck | TEM_FI1; KJ930397 |  | FI17 |
| FI17.2 | Mt Finnigan | Parrots Ck | TEM_FI3; KJ930399 |  | FI17 |
| FI17.3 | Mt Finnigan | Parrots Ck | TEM_FI1; KJ930397 |  | FI17 |
| FI17.4 | Mt Finnigan | Parrots Ck | TEM_FI3; KJ930399 |  | FI17 |
| FI18.1 | Mt Finnigan | Parrots Ck | TEM_FI4; KJ930396 |  | FI18 |
| FI19.1 | Mt Finnigan | Annan Ck | TEM_FI5; KJ930400 |  | FI19 |
| FI19.2 | Mt Finnigan | Annan Ck | TEM_FI3; KJ930399 |  | FI19 |
| FI19.3 | Mt Finnigan | Annan Ck | TEM_FI3; KJ930399 |  | FI19 |
| FI19.4 | Mt Finnigan | Annan Ck | TEM_FI3; KJ930399 |  | FI19 |
| FI20.1 | Mt Finnigan | Annan Ck | TEM_FI1; KJ930397 |  | F20* |
| FI20.2 | Mt Finnigan | Annan Ck | TEM_FI1; KJ930397 |  | F20* |
| FI4.1 | Mt Finnigan | Annan Ck | TEM_FI3; KJ930399 |  | FI4 |
| FI4.2 | Mt Finnigan | Annan Ck | TEM_FI3; KJ930399 |  | FI4 |
| FI4.3 | Mt Finnigan | Annan Ck | TEM_FI3; KJ930399 |  | FI4 |
| FI7.1 | Mt Finnigan | Annan Ck | TEM_FI3; KJ930399 | TEM_1; KJ941013 | FI7 |
| FI7.2 | Mt Finnigan | Annan Ck | TEM_FI3; KJ930399 |  | FI7 |
| FI7.3 | Mt Finnigan | Annan Ck | TEM_FI3; KJ930399 |  | FI7 |
| PB36.1 | Mt Pieter Botte | R.Meg River | TEM_PB1; KJ930401 |  | PB36 |
| PB36.12 | Mt Pieter Botte | R.Meg River | TEM_PB1; KJ930401 |  | PB36 |
| PB36.13 | Mt Pieter Botte | R.Meg River | TEM_PB1; KJ930401 |  | PB36 |
| PB36.2 | Mt Pieter Botte | R.Meg River | TEM_PB1; KJ930401 | TEM_2; KJ941014 | PB36 |
| PB36.3 | Mt Pieter Botte | R.Meg River | TEM_PB1; KJ930401 |  | PB36 |
| PB36.5 | Mt Pieter Botte | R.Meg River | TEM_PB1; KJ930401 |  | PB36 |
| PB36.6 | Mt Pieter Botte | R.Meg River | TEM_PB1; KJ930401 |  | PB36 |
| PB36.7 | Mt Pieter Botte | R.Meg River | TEM_PB1; KJ930401 |  | PB36 |
| PB36.8 | Mt Pieter Botte | R.Meg River | TEM_PB1; KJ930401 |  | PB36 |
| PB36.9. | Mt Pieter Botte | R.Meg River | TEM_PB1; KJ930401 |  | PB36 |
| PB38.1 | Mt Pieter Botte | R.Meg River | TEM_PB1; KJ930401 | TEM_2; KJ941014 | PB38 |
| PB38.11 | Mt Pieter Botte | R.Meg River | TEM_PB1; KJ930401 |  | PB38 |
| PB38.2 | Mt Pieter Botte | R.Meg River | TEM_PB1; KJ930401 | TEM_2; KJ941014 | PB38 |
| PB40.1 | Mt Pieter Botte | R.Meg River | TEM_PB2; KJ930402 |  | PB40 |
| 509R _D Blair | Mount Finnigan | Horan’s Ck | TEM_TP10; KJ930412 |  | Unknown |
| *Temnosewellia aphyodes -* OUTGROUP | Mt Lewis | Leichhardt Ck | 530FR; KJ958928 |  | *E. fleckeri* |
| TP24.1 | Thornton Peak | Hilda Ck | TEM_TP7; KJ930409 |  | TP24* |
| TP24.2 | Thornton Peak | Hilda Ck | TEM_TP2; KJ930404 |  | TP24* |
| TP24.3 | Thornton Peak | Hilda Ck | TEM_TP2; KJ930404 |  | TP24* |
| TP24.4 | Thornton Peak | Hilda Ck | TEM_TP3; KJ930405 |  | TP24* |
| TP24.5 | Thornton Peak | Hilda Ck | TEM_TP2; KJ930404 |  | TP24* |
| TP24.6 | Thornton Peak | Hilda Ck | TEM_TP8; KJ930410 |  | TP24* |
| TP24.7 | Thornton Peak | Hilda Ck | TEM_TP2; KJ930404 |  | TP24* |
| TP26.1 | Thornton Peak | Hilda Ck | TEM_TP2; KJ930404 |  | TP26* |
| TP28.1 | Thornton Peak | Hilda Ck | TEM_TP9; KJ930411 |  | TP28 |
| TP28.2 | Thornton Peak | Hilda Ck | TEM_TP2; KJ930404 |  | TP28 |
| TP28.6 | Thornton Peak | Hilda Ck | TEM_TP7; KJ930409 |  | TP28 |
| TP31.1 | Thornton Peak | Hilda Ck | TEM_TP3; KJ930405 | TEM_2; KJ941014 | TP31 |
| TP31.3 | Thornton Peak | Hilda Ck | TEM_TP4; KJ930406 |  | TP31 |
| TP31.4 | Thornton Peak | Hilda Ck | TEM_TP3; KJ930405 |  | TP31 |
| TP31.5 | Thornton Peak | Hilda Ck | TEM_TP4; KJ930406 |  | TP31 |
| TP32.1 | Thornton Peak | Hilda Ck | TEM_FI4; KJ930396 |  | TP32 |
| TP32.2 | Thornton Peak | Hilda Ck |  | TEM_1; KJ941013 | TP32 |
| TP33.1 | Thornton Peak | Hilda Ck | TEM_TP5; KJ930407 |  | TP33 |
| TP33.2 | Thornton Peak | Hilda Ck | TEM_TP6; KJ930408 |  | TP33 |
| TP34.1 | Thornton Peak | Hilda Ck | TEM_TP1; KJ930403 | TEM_1; KJ941013 | TP34 |
| TP34.2 | Thornton Peak | Hilda Ck | TEM_FI1; KJ930397 |  | TP34 |
